# Neuromyelitis optica in a pregnant woman with systemic lupus erythematous: A case report

**Published:** 2016-10-07

**Authors:** Fariborz Khorvash, Nafiseh Esmaeil, Omid Mirmosayyeb, Nahid Eskandari, Homayoon Salimian

**Affiliations:** 1 Department of Neurology, Neurosciences Research Center, Alzahra Hospital, Isfahan University of Medical Sciences, Isfahan, Iran; 2 Department of Immunology, School of Medicine, Isfahan University of Medical Sciences, Isfahan, Iran; 3 Neurosciences Research Center, Alzahra Hospital, Student Research Committee, Isfahan University of Medical Sciences, Isfahan, Iran; 4 School of Medicine, Shahrekord University of Medical Sciences, Shahrekord, Iran

**Keywords:** Systemic Lupus Erythematosus, Neuromyelitis Optica, Pregnant Woman

Systemic lupus erythematosus (SLE) is an autoimmune disease which predominantly affects women of childbearing age. Likewise, patients suffering from SLE are at a higher risk of developing other autoimmune diseases.

Neuromyelitis optica (NMO), is a rare inflammatory demyelinating affliction of the central nervous system (CNS) principally characterized by recurrent optic neuritis (ON) and longitudinal extensive transverse myelitis (LETM).^1^

We report a case of a 30-year-old woman at 6 weeks of her second pregnancy with a three-year history of controlled SLE that was subsequently identified to have a NMO.

She had been presenting a dexter paraplegia from inception of pregnancy. Her unexpected numbness primarily had started in both lower extremities (especially right leg) and then, increasingly had progressed to the waist with arthralgia for 5 months. Visual evoked potential (VEP) was performed, by virtue of her complaints of blurred vision and abnormal VEP in both eyes. She had denied headache, dizziness, diplopia, fever and no history of respiratory, urinary and gastrointestinal problems. Moreover, she had a discoid rash on her right cheek. 

On physical examination, she was oriented, well-nourished and her cranial nerves were intact. Besides, on musculoskeletal assessment, arthritis symptoms were not recognized though she was slow to perform tasks. She had a revealing enervation in all extremities and her motor strength was at a rate of 2/5. Upper motor neuron dysfunction was suggested according to positive Babinski sign and superficial abdominal hyporeflexia. 

Routine blood tests and chemistry analysis including complete blood count (CBC), thyroid stimulating hormone and coagulation tests were unremarkable. Autoimmune markers such as rheumatoid factor, lupus anti-coagulant and anti-phospholipid antibodies including anti-cardiolipin and anti-2-glycoprotein were negative but her anti-Sjögren's-syndrome-related antigen-A (anti-SS-A) test, anti-nuclear antibody (ANA) and NMO-IgG test were positive. She had decreased level of serum complement factor; C3 and T4 levels were raised in assessment of thyroid function. 

Normal magnetic resonance imaging (MRI) of the brain was seen without plaques. Thoracic cord MRI revealed hyperintense signal lesions from cervical segment 5 to thoracic segment 4 (7 hyperintense demyelinated segments). In addition, hyperintense signal at the thoracic spine level with holocord involvement was seen. According to patient’s history (discoid rash), physical examination results (paraplegia), autoimmune tests (positive NMO-IgG) and imaging findings (LETM), a diagnosis of NMO secondary to an acute SLE flair was formed.

The patient was treated with 400 mg of oral hydroxychloroquine once a day for 3 months, and she had no further cutaneous or articular symptoms. At the 8^th^ week of gestation, she had abortion and three pulses of intravenous methyl-prednisolone and daily pulse of cyclophosphamide were administered. Since her condition had not improved after receiving therapy, four sessions of plasmapheresis with 3 liters volume and fresh frozen plasma (FFP) replacement was done. Significant improvement was observed after plasmapheresis, and the patient limb’s motility recurred. 

SLE is a multisystem autoimmune disease, which its pathophysiology may have consequences on all components of the CNS. The CNS and peripheral nervous systems (PNS) may be involved in SLE. It is well known that NMO is closely linked with other autoimmune diseases; however it has been barely reported in patients with SLE. Our case has an extensive role in justifying the fact that the contact between SLE and NMO can happen during the life span. Mula et al. have hypothesized that early treatment, by combination of plasmapheresis and immunosuppressive agents, may be related to more beneficial conclusion in patients with SLE and longitudinal myelitis (LM).^2^ In a similar case report study, a patient with 19 myelitis attacks was reported during rituximab therapy.^3^ However, our case experienced one myelitis attack from her NMO onset that substantially improved after plasmapheresis with 6 months follow-up. 

From the immunological perspective, cell-mediated immunity converts to increased humoral immunity in normal pregnancy. Pregnancy tends to aggravate NMO and SLE which are B-cell-mediated autoimmune diseases. On the other hand, previous studies declare that NMO-IgG can damage placenta and cause fetal death in mice.^4^

A study reported a case of SLE in 28-year-old women, who diagnosed NMO in the fourth month of her pregnancy. In that case, the patient did not have such a favorable consequence.^5^ Therefore, since SLE-associated NMO generally has a poor prognosis and possibly prompting disability and may be the cause to abortion, early diagnosis seems essential. 

In conclusion, considering the fact that the incidence of NMO in an underlying autoimmune connective tissue disease represents a co-occurrence of two autoimmune disorders, pregnancy could be suggested as a trigger for this concurrency. Eventually as occurrence of NMO in patients with SLE, especially in pregnancy, is extraordinary, specific researches with collaboration from several groups would be invaluable in order to overcome the limitation of papers such as this one.

## Notes:

**How to cite this article:** Khorvash F, Esmaeil N, Mirmosayyeb O, Eskandari N, Salimian H. Neuromyelitis optica in a pregnant woman with systemic lupus erythematous: A case report. Iran J Neurol 2016; 15(4): 232-4.
